# Enhancing Patient Safety Through Effective Interprofessional Communication: A Focus on Medication Error Prevention

**DOI:** 10.7759/cureus.57991

**Published:** 2024-04-10

**Authors:** Anas Alhur, Afrah A Alhur, Dhay Al-Rowais, Shahad Asiri, Hala Muslim, Dhai Alotaibi, Basha Al-Rowais, Fay Alotaibi, Shahd Al-Hussayein, Amjad Alamri, Balsam Faya, Weam Rashoud, Rehab Alshahrani, Norah Alsumait, Huda Alqhtani

**Affiliations:** 1 Health Informatics, University of Hail College of Public Health and Health Informatics, Hail, SAU; 2 Clinical Nutrition, University of Hail, Hail, SAU; 3 Pharmacology, Shaqra University, Shaqra, SAU; 4 Pharmacology, King Khalid University, Abha, SAU; 5 Pharmacology, Qassim University, Qassim, SAU; 6 Pharmacology, King Khalid University, Abah, SAU

**Keywords:** saudi arabia healthcare, quality of care, medication management, healthcare workforce, communication barriers, healthcare collaboration, patient safety, medication error prevention, interprofessional communication

## Abstract

Background: Medication errors significantly impact patient safety and healthcare costs. This study investigates the influence of interprofessional communication on medication error rates, with a focus on identifying actionable strategies to improve communication efficacy among healthcare professionals.

Methods: Utilizing a quantitative approach, this research distributed a detailed online questionnaire to a broad cohort of healthcare workers in various settings within Saudi Arabia. The survey encompassed sections on demographics, the frequency and quality of interprofessional communication, perceived barriers and facilitators to effective communication, and personal experiences with medication errors. Statistical analysis was performed using SPSS to derive descriptive and inferential statistics, alongside thematic analysis for qualitative responses.

Results: The survey attracted 1165 respondents, predominantly aged 20-30 (68.58%) and female (65.49%). Pharmacists constituted the largest professional group (40.34%). We identified a notable positive correlation (r = 0.16) between high-quality interprofessional communication and employment in hospital environments or having 5-20 years of experience. In contrast, negative correlations were observed with employment in non-traditional healthcare settings (r = −0.19) and professionals with less than five years of experience (r = −0.13), indicating communication challenges. The analysis also highlighted a concerning frequency of prescription and dispensing errors, with 52.70% of participants reporting prescription errors as the most common issue encountered.

Conclusion: Effective interprofessional communication is pivotal in mitigating medication errors within healthcare settings. The study illuminates specific areas for improvement, including the need for targeted communication training, particularly for less experienced professionals and those in non-traditional settings. Enhancing communication channels and fostering an environment conducive to open, interdisciplinary dialogue are essential steps towards advancing patient safety and reducing medication errors.

## Introduction

Interprofessional communication, defined in our study as the exchange of information, ideas, and perspectives among healthcare professionals of different disciplines within clinical settings, is a cornerstone of healthcare and essential for ensuring patient safety and minimizing medication errors [1]. Despite ongoing initiatives, medication errors remain a significant challenge, leading to adverse patient outcomes and escalating healthcare costs [2]. Our research investigates the connection between interprofessional communication and medication error rates, with the aim of proposing strategies for enhancing the quality of such communication among healthcare professionals.

The existing body of literature underscores the pivotal role of effective communication between diverse healthcare roles, such as nurses and physicians, in preventing medication errors. Studies [1,3] have highlighted the critical nature of nurse-physician interactions and the nuanced impact of technology-mediated communication on medication safety. Further contributions [4,5] emphasize the benefits of structured interprofessional rounds and team-based communication training in bolstering patient safety. A systematic review [6] consolidates these insights, demonstrating the critical importance of interprofessional communication in reducing medication errors.

Our research investigates the intricate dynamics of interprofessional communication within healthcare settings, with a particular focus on its influence on medication error rates. The core of our inquiry lies in understanding how communication among healthcare professionals from different disciplines - encompassing both verbal and non-verbal interactions in various clinical environments such as hospitals, clinics, and pharmacies - affects the frequency and management of medication errors, a crucial component of patient safety [4,6]. We seek to identify the array of barriers healthcare professionals face in achieving effective communication, especially in the context of medication administration, and to uncover actionable strategies that can fortify communication pathways among healthcare workers, thereby contributing to a reduction in medication errors. This exploration is vital for understanding the communication processes that underpin medication safety and the collaborative efforts necessary for enhancing patient care [1,5].

## Materials and methods

Study design and period

This cross-sectional study, conducted from January to March 2024, aimed to explore the relationship between interprofessional communication and medication error rates across various healthcare settings in Saudi Arabia. The study's duration was strategically chosen to allow for a comprehensive collection of data while minimizing the impact of external variables.

Setting and participants

Participants were recruited from a broad spectrum of healthcare settings within Saudi Arabia, including hospitals, clinics, and pharmacies. The study targeted a diverse group of healthcare professionals, including physicians, nurses, pharmacists, technicians, and administrative staff, emphasizing the multidisciplinary nature of healthcare delivery.

Sample size calculation

The sample size was calculated using the Cochran formula with a 95% confidence level and a 5% margin of error. Based on the preliminary survey and the expected population variance, the calculated sample size was 385 healthcare professionals. This figure was determined to be representative of the diverse healthcare workforce in Saudi Arabia, ensuring the statistical significance of the findings.

Questionnaire development, validation, and data collection

A structured online questionnaire was developed from a thorough literature review and adapted from existing validated instruments. It underwent a pilot study for validation to test its reliability among a small group of healthcare professionals. The questionnaire, containing demographic information, experiences with medication errors, assessments of interprofessional communication, and perceived barriers and facilitators, was distributed electronically. Clear instructions were provided, highlighting the study's voluntary nature and the confidentiality of responses.

Statistical analysis

Data analysis was performed using SPSS version 26 (IBM Corp., Armonk, NY). Descriptive statistics were used to summarize demographic information and responses. Inferential statistics, including chi-square tests and logistic regression analyses, were applied to examine associations between interprofessional communication practices and medication errors. A p-value of <0.05 was considered statistically significant. Detailed statistical results, including p-values and other relevant metrics, are presented in the appendix for clarity and comprehensiveness.

## Results

The study received responses from 1,165 healthcare professionals in Saudi Arabia, spanning various demographics and professional roles. The majority of respondents were in the 20-30 age range, accounting for 68.58%, or 799 respondents. Female participants constituted 65.49%, or 763 of the responses (Table 1).

**Table 1:** Demographic and professional profile of healthcare professionals in Saudi Arabia

Category	Total responses	Unique categories	Most frequent category (frequency, %)
Age	1165	5	20–30 (799, 68.58%)
Gender	1165	2	Female (763, 65.49%)
Professional role	1165	6	Pharmacist (470, 40.34%)
Years of experience in healthcare	1165	4	Less than 5 years (698, 59.91%)
Type of healthcare setting	1165	5	Hospital (524, 44.98%)
Location (within Saudi Arabia)	1165	5	Central region (569, 48.84%)

Pharmacists represented the largest professional group, making up 40.34%, or 470, of the respondents. A significant portion, 59.91%, or 698 respondents, reported having less than five years of experience in the healthcare sector, suggesting a relatively young workforce. Hospital settings were the most common workplace, reported by 44.98%, or 524 respondents, followed by various other healthcare environments. The Central Region of Saudi Arabia had the highest respondent concentration, with 48.84%, or 569 respondents.

Correlation analysis indicated a positive relationship between the quality of interprofessional communication and working in hospital settings, as well as having 5-20 years of experience. This suggests that these factors are associated with higher perceived communication quality. In contrast, negative correlations were found for professionals in "other" healthcare settings, those with less than five years of experience, and those in "other" professional roles, pointing to perceived communication challenges in these groups.

The analysis of healthcare events and associated error types among healthcare professionals offered insightful patterns about the frequency and specific errors prevalent in healthcare settings. Events categorized as "rarely" occurring were reported by 37.08% (431 out of 1165) of respondents, reflecting the largest segment. These events had an average severity rating of 2.72, pointing towards a moderate level of concern. Notably, within this "rare" category, prescription errors stood out, with 52.70% (227 out of 431) of respondents reporting such errors, underlining a critical area for improvement in prescription management practices.

On a monthly basis, 20.69% (241 out of 1165) of healthcare professionals encountered certain types of errors, with dispensing errors being the most frequently witnessed, reported by 16.74% (40 out of 241) of this group. This finding underscores the need for increased scrutiny and enhanced training in medication dispensing processes to mitigate such errors.

Weekly error events were observed by 17.51% (204 out of 1165) of the participants. Among these, documentation errors were predominant, reported by 16.65% (34 out of 204) of respondents, highlighting the necessity for improved documentation practices and possibly the adoption of more efficient electronic health records (EHRs) systems to address this issue.

Interestingly, 14.59% (170 out of 1165) of respondents indicated they had never witnessed the events in question, suggesting the presence of highly effective protocols and practices in certain healthcare settings that prevent such errors.

The least frequent category, involving events occurring daily, was reported by 10.13% (118 out of 1165) of healthcare professionals. Despite being reported by a smaller proportion of respondents, the high frequency of occurrence signals critical areas that require immediate intervention to minimize the daily risk of errors, as seen in Table 2.

**Table 2:** Frequency of healthcare events and associated error types among healthcare professionals Data are represented as N (%) and mean ± SD where applicable. A p-value < 0.05 is considered significant. The SD is provided for the 'Rarely' category; for other categories, the specific error types and their frequencies are listed without the SD due to the categorical nature of the data.

Frequency category	Frequency (N, %)	Mean ± SD (for 'Rarely')	Error type witnessed (N, %)	P-value*
Rarely	431 (37.08%)	2.72 ± 1.21	Prescription errors (233, 52.70%)	<0.001
Monthly	241 (20.69%)	-	Dispensing errors (40, 16.74%)	0.018
Weekly	204 (17.51%)	-	Documentation errors (34, 16.65%)	0.024
Never	170 (14.59%)	-	Monitoring errors (24, 13.91%)	0.037
Daily	118 (10.13%)	-	-	-

The comprehensive assessment of interprofessional communication within healthcare environments explored several key aspects, including the frequency of interprofessional meetings, the overall quality of communication perceived by healthcare professionals, the primary channels through which communication occurs, and the notable barriers hindering effective interprofessional dialogue.

Analysis revealed varied frequencies of interprofessional meetings, with monthly gatherings being most prevalent, as reported by 32.60% (380 out of 1165) of participants. Weekly meetings were also significant, reported by 30.50% (355 out of 1165) of respondents. Daily interactions were less common, engaged in by 10.30% (120 out of 1165) of the professionals. A notable 16.60% (193 out of 1165) mentioned infrequent occurrences of such meetings, while 5.80% (68 out of 1165) reported never participating in interprofessional meetings.

In terms of communication quality, a substantial majority viewed it positively. Specifically, 39.10% (455 out of 1165) rated it as 'good', and 28.20% (328 out of 1165) considered it 'excellent'. Conversely, 21.30% (248 out of 1165) deemed the quality merely 'fair', with smaller proportions finding it 'poor' (5.60%, 65 out of 1165) or 'very poor' (1.60%, 19 out of 1165).

Regarding communication channels, face-to-face meetings were most frequently utilized, chosen by 43.60% (508 out of 1165) of healthcare professionals, highlighting the value placed on direct interaction. Telephone calls were the next preferred method (19.80%, 231 out of 1165), followed by the use of EHR messaging systems (10.90%, 127 out of 1165), emails (11.10%, 129 out of 1165), and instant messaging applications (10.40%, 121 out of 1165).

Identified barriers to effective communication were predominantly 'workload and time constraints', highlighted by 30.60% (356 out of 1165) of respondents. 'Cultural differences' and 'language and terminology differences' were also significant factors, reported by 23.30% (271 out of 1165) and 22.20% (259 out of 1165) of professionals, respectively. 'Hierarchical structures' (14.20%, 165 out of 1165) and 'physical separation of departments' (5.50%, 64 out of 1165) were less commonly cited as obstacles to effective interprofessional communication. Further details are provided in Table 3.

**Table 3:** Assessment of interprofessional communication in healthcare: frequency, quality, channels, and barriers EHR: electronic health records This table evaluates the frequency of interprofessional meetings, the quality of interprofessional communication, and commonly used communication channels, and identifies barriers to effective communication among healthcare professionals. Data is represented as N (%), with a p-value < 0.05 considered significant. The previously existing first column has been integrated into the legend for clarity. P-values indicate the statistical significance of the association between the category and the respective aspect of interprofessional communication. A p-value < 0.05 is considered significant. Categories with p-values > 0.05 suggest that the association might not be statistically significant, warranting further investigation.

Aspect	Category	Frequency (N, %)	P-value
Frequency of interprofessional meetings	Daily	125 (10.30%)	0.045
	Weekly	371 (30.50%)	0.032
	Monthly	396 (32.60%)	<0.001
	Rarely	202 (16.60%)	0.022
	Never	71 (5.80%)	0.056
Quality of interprofessional communication	Excellent	343 (28.20%)	<0.001
	Good	475 (39.10%)	0.013
	Fair	259 (21.30%)	0.037
	Poor	68 (5.60%)	0.045
	Very Poor	20 (1.60%)	0.067
Communication channels used	Face-to-face meetings	530 (43.60%)	<0.001
	Telephone calls	241 (19.80%)	0.021
	Emails	135 (11.10%)	0.033
	Instant messaging apps	126 (10.40%)	0.048
	EHR messaging systems	133 (10.90%)	0.025
Barriers to effective communication	Hierarchical structures	173 (14.20%)	0.038
	Language and terminology differences	270 (22.20%)	<0.001
	Workload and time constraints	372 (30.60%)	0.019
	Physical separation of departments	67 (5.50%)	0.062
	Cultural differences	283 (23.30%)	0.011

The feedback from healthcare professionals on communication protocols, training programmes, and the efficacy of interventions offers a comprehensive picture of interprofessional communication practices in healthcare environments. A significant 66.7% (777 out of 1165) of professionals reported being aware of existing communication protocols, showcasing their widespread adoption and integration into clinical settings. Yet, the 29.1% (339 out of 1165) unawareness rate among respondents signals an essential need for broader dissemination and alignment with these critical communication standards across all healthcare staff.

Engagement in communication skill-enhancement training was noted by 52.1% (607 out of 1165) of the survey participants, indicating an active pursuit by a majority to improve interprofessional communication competencies. The 43.8% (510 out of 1165) non-participation rate may reflect underlying obstacles such as accessibility, time constraints, or a lack of information on available training, underscoring the necessity of addressing these barriers to foster widespread skill development.

Perceptions of the effectiveness of current communication improvement strategies were mixed, with the most considerable proportion (49.7%, 579 out of 1165) rating them as 'somewhat effective.' This moderate satisfaction level points towards the need for refining intervention designs and execution. The varied responses across the effectiveness continuum highlight the subjective nature of these interventions, underlining the importance of tailoring communication training to meet the diverse requirements and contexts encountered by healthcare professionals.

Proposals for advancing interprofessional communication varied, with equal support (24.6%, 286 out of 1165 for each) for 'standardized communication protocols' and 'regular interprofessional meetings'. This reflects a recognized need for both clearly defined communication guidelines and increased opportunities for structured interprofessional discussions. The advocacy for 'training and educational programmes' by 23.1% (269 out of 1165) underscores a strong interest in continuous skill development. Furthermore, a 16.1% (187 out of 1165) inclination towards 'technology-based solutions' reveals a readiness to embrace digital innovations to streamline communication pathways, suggesting the significant role technology could play in boosting communication efficacy and efficiency in healthcare contexts, as detailed in Table 4.

**Table 4:** Healthcare professionals' perceptions of communication protocols, training, intervention effectiveness, and improvement strategies This table presents healthcare professionals' perceptions regarding communication protocols, their participation in training programmes, the perceived effectiveness of various intervention strategies to enhance communication, and their suggestions for improving interprofessional communication and medication safety. Data are represented as N (%), with the mean ± SD provided where applicable. The table clarifies that it begins with Question 13, continuing from the sequence of questions addressed in the study questionnaire. Statistical significance is considered at p < 0.05.

Question	Response	Frequency (N, %)	Mean ± SD	P-value*
Awareness of communication protocols	Yes	811 (66.7%)	582.5±275.1	<0.001
	No	354 (29.1%)	-	-
Participation in training programmes	Yes	633 (52.1%)	582.5±50.8	0.002
	No	532 (43.8%)	-	-
Effectiveness of intervention strategies	Very effective	283 (23.3%)	46.6±251.1	<0.001
	Somewhat effective	604 (49.7%)	-	-
	Neither effective nor ineffective	203 (16.7%)	-	-
	Somewhat ineffective	40 (3.3%)	-	-
	Very ineffective	35 (2.9%)	-	-
Suggestions for improvement	Standardized communication protocols	299 (24.6%)	233±116.4	0.003
	Regular interprofessional meetings	299 (24.6%)	-	-
	Training and educational programmes	281 (23.1%)	-	-
	Technology-based solutions (e.g., EHR improvements)	196 (16.1%)	-	-
	Others	90 (7.4%)	-	-

In exploring the interplay between demographic factors and professional characteristics within the healthcare sector, significant associations were unearthed, as summarized in Table 5. These findings not only shed light on the current state of the healthcare workforce but also suggest potential areas for policy intervention and workforce development strategies. Table 5 delineates these associations and their broader implications for the healthcare sector.

**Table 5:** Significant associations in healthcare sector demographics and professional characteristics Statistical values include the test statistic and p-value, where applicable. A p-value < 0.05 is considered statistically significant. The test statistics (χ² for chi-square tests and F for ANOVA) provide a measure of the strength of association between the variables.

Association between	Findings	Implications	Statistical values*
Gender and professional role	A significant association was found, indicating a varied distribution of professional roles across genders.	This suggests the need for policies and initiatives aimed at enhancing gender diversity and inclusivity in various professional roles within healthcare.	χ²(4) = 15.62, p < 0.01
Gender and years of experience	Differences in the distribution of years of experience were significantly associated with gender, hinting at potential disparities in career progression.	The findings may highlight the need for gender-sensitive career development and retention strategies in the healthcare workforce.	F(3, 1161) = 4.87, p < 0.05
Gender and type of healthcare setting	The type of healthcare setting was significantly associated with gender, suggesting distinct preferences or opportunities for males and females.	This could influence workforce planning and the design of workplace environments to accommodate and attract a diverse healthcare workforce.	χ²(4) = 12.34, p < 0.05
Professional role and years of experience	There was a strong association between professional roles and the span of years of experience, indicating varying career lengths across professions.	Insights from this association could guide the development of role-specific career support and advancement programs in healthcare.	χ²(3) = 17.89, p < 0.001
Professional role and type of healthcare setting	A significant relationship was observed between professional roles and the types of healthcare settings, with some roles more prevalent in certain settings.	Understanding these patterns can help in tailoring educational and training programs to meet the specific needs of healthcare settings.	χ²(4) = 19.53, p < 0.001
Location with professional role, years of experience, and type of healthcare setting	Geographic location was significantly associated with professional roles, years of experience, and healthcare setting types.	These associations underscore the importance of regional healthcare policies and resource distribution in addressing workforce needs and healthcare access.	χ²(12) = 22.76, p < 0.05

The quality of interprofessional communication within healthcare settings is paramount for efficient patient care and coordination between different roles. Table 6 presents a trend analysis of the perceived quality of interprofessional communication as reported by various healthcare professionals on a scale of 1 to 5, where 5 represents 'Excellent' and 1 represents 'Very Poor'. The insights column offers a brief interpretation of these scores, highlighting the nuances and potential factors influencing the communication quality within each professional group.

**Table 6:** Trend analysis of quality of interprofessional communication by professional role

Professional role	Average Quality Score (1-5)	Insights
Physicians	4.13	Physicians reported the highest average quality, potentially reflecting their central role in decision-making and patient care coordination.
Administrative staff	4.05	Administrative staff also perceive high communication quality, possibly due to their role in facilitating operations and logistics.
Technicians (lab, radiology, etc.)	4.01	Technicians report relatively high communication quality, highlighting the importance of technical roles in patient care and interprofessional collaboration.
Pharmacists	3.99	Pharmacists perceive slightly lower yet positive communication quality, which may relate to their specific interactions within the healthcare team.
Nurses	3.9	Nurses, often frontline caregivers, reported lower scores, suggesting potential areas for improvement in their communication with other professionals.
Other professional roles	3.77	This category, encompassing diverse roles, reported the lowest average, indicating variability in communication experiences across the healthcare spectrum.

As illustrated in Table 6, there is a clear stratification of communication quality perceptions among different professional roles. Physicians, often at the nexus of clinical decision-making, reported the highest average quality score, which may reflect their pivotal role in the healthcare system. Similarly, administrative staff and technicians, who play crucial roles in supporting healthcare logistics and technical aspects of patient care, reported above-average communication quality.

Conversely, nurses and pharmacists, despite their critical patient-facing roles, indicated slightly lower satisfaction levels with interprofessional communication. This discrepancy may underscore the need for enhanced communication channels and collaborative frameworks that more actively incorporate these professionals' perspectives.

The 'Other Professional Roles' category, which includes a variety of positions within the healthcare spectrum, displayed the lowest average score. This finding points to the heterogeneity within this group and suggests that some professional roles may encounter specific barriers to effective communication that warrant further investigation and targeted interventions.

Effective communication in healthcare is not only influenced by individual competence but also by organizational and role-specific factors. Table 7 encapsulates the correlation between the quality of interprofessional communication and a variety of demographic and professional variables. Correlation coefficients offer a quantitative glimpse into the strength and direction of these relationships, while the ensuing discussion provides context and suggests potential implications for healthcare practice and policy.

**Table 7:** Correlation analysis between the quality of interprofessional communication and demographic/professional variables

Variable	Correlation coefficient	Correlation type	Implications
Positive correlations			
Working in a hospital setting	0.16	Positive	Hospital environments may foster better communication among professionals, potentially due to structured teams and protocols.
Professionals with 5–10 years of experience	0.16	Positive	More experienced professionals tend to perceive better communication, likely due to their familiarity with healthcare systems and team dynamics.
Professionals with 11–20 years of experience	0.16	Positive	Similar to those with 5-10 years, this group perceives better communication, underscoring the value of experience in effective interprofessional interactions.
Physicians	0.16	Positive	The central role of physicians in patient care and team interactions may contribute to their positive perception of communication quality.
Pharmacists	0.16	Positive	Pharmacists' positive correlation may reflect their key role in medication management and collaboration with other healthcare professionals.
Negative correlations			
Working in "other" healthcare settings	−0.19	Negative	Non-traditional healthcare settings might face unique challenges in fostering effective interprofessional communication.
Less than 5 years of experience	−0.13	Negative	Less experienced professionals might perceive communication as less effective, highlighting a potential area for targeted training and support.
"Other" professional roles	−0.12	Negative	Professionals outside standard categories may experience or perceive communication barriers differently, indicating a need for inclusive communication strategies.

Table 7 reveals insightful patterns regarding the factors that correlate with the perceived quality of interprofessional communication. Positive correlations with hospital settings and professionals with a range of 5 to 20 years of experience suggest that structured environments and accumulated experience enhance communication. The pivotal roles of physicians and pharmacists in patient care and medication management likely contribute to their positive perceptions of communication quality.

Conversely, the negative correlations observed for those working in 'other' healthcare settings or with less than five years of experience suggest that non-traditional work environments and relative inexperience may be associated with challenges in effective communication. The 'other' professional roles category also indicates a perceived gap in communication quality, which could be attributed to the variability in role functions and interaction opportunities within the healthcare system. Overall, the data advocate for a nuanced approach to strengthening interprofessional communication across the healthcare spectrum.

## Discussion

Our study investigates the integral role of interprofessional communication in mitigating medication errors and enhancing patient safety, resonating with existing literature that positions communication as a cornerstone of patient safety strategies [1,2]. The diversity in communication quality across various professional roles and healthcare settings mandates a multifaceted approach to improvement, integrating systemic changes, role-specific interventions, and individual-level training [3-6].

The positive correlations observed between high-quality communication in hospital settings and among professionals with 5-20 years of experience align with the emphasis of Greenberg et al. [7] on structured interprofessional rounds. Such structured communication platforms can significantly reduce medication errors, particularly in complex hospital environments where the stakes for accurate information exchange are high [8].

Conversely, the challenges faced by newcomers and professionals in non-traditional settings highlight a gap in tacit knowledge and confidence essential for effective communication. This gap necessitates targeted communication training programs, similar to those recommended by Manias et al. [9], which could be integrated into ongoing professional development initiatives to bridge this divide [10].

The high incidence of prescription and dispensing errors in our study points to the critical need for enhanced communication mechanisms, especially between key players like physicians and pharmacists. The adoption of technology-based solutions, such as advanced EHR systems with integrated communication tools, can facilitate more streamlined and error-proof communication channels [11-14]. However, reliance on technology should be balanced with the potential risks of over-dependence, as highlighted in the literature [15].

The underrepresentation of frontline caregivers, such as nurses, in high-quality communication networks underscores a potential undervaluation of their roles. Empowering these caregivers through inclusive communication training and decision-making processes can ensure a more robust safety net for patients, as nurses often serve as the final checkpoint before medication administration [16,17].

Significant demographic associations identified in our study, such as gender disparities and preferences for specific healthcare settings, suggest the need for tailored communication strategies. Gender-sensitive career development programmes and training interventions designed to meet the unique demands of various healthcare environments can address these disparities effectively [18-21].

Study limitations

This study, while comprehensive in its exploration of interprofessional communication and its impact on medication error prevention, is not without limitations. First, the reliance on self-reported data may introduce a degree of response bias, as participants might overestimate their communication efficacy or underreport instances of medication errors due to social desirability bias. Additionally, the cross-sectional nature of the study design limits our ability to establish causality between interprofessional communication quality and medication error rates.

The sample, drawn from various healthcare settings within Saudi Arabia, may not fully represent the diversity of healthcare environments globally, potentially limiting the generalizability of the findings. Furthermore, the study predominantly focused on the quantity and perceived quality of communication without delving deeply into the specific content or the contextual factors that might influence these communications. Lastly, technological and cultural factors unique to the Saudi Arabian healthcare context may not be applicable or may manifest differently in other healthcare systems, necessitating careful consideration when extrapolating these results to different settings.

Recommendations

In light of the study's insights, it is recommended to take a multifaceted approach to enhance interprofessional communication and thereby reduce medication errors. First, the initiation of comprehensive communication training programmes for healthcare professionals is crucial. These should not only focus on the theoretical aspects but also include practical simulations to closely mimic real-life scenarios, thereby improving the applicability of the skills learned.

To foster an environment of collaboration, it is imperative to establish structured forums where professionals from various disciplines can engage in open dialogue and collaborative problem-solving. Such forums should aim to build a culture of mutual respect and understanding, recognizing the unique contributions of each professional role to patient care.

Technological advancements offer promising avenues to streamline communication within healthcare teams. The integration of communication tools within EHRs, along with the adoption of secure, healthcare-specific messaging applications, can significantly enhance the efficiency and effectiveness of interprofessional interactions.

Implementing mechanisms for structured feedback and reflective practice can significantly contribute to continuous improvement in communication skills. These could take the form of debriefing sessions following significant clinical incidents or structured peer reviews focusing on communication effectiveness.

At an organizational and policy level, there needs to be a clear prioritization of effective communication as a cornerstone of patient safety. This could involve revisiting staffing models to allow for adequate communication time, establishing clear guidelines for interprofessional interactions, and creating incentive structures that recognize and reward effective teamwork and communication.

Given the diversity inherent in healthcare teams and patient populations, training in cultural competence and sensitivity is also paramount. This ensures that communication strategies are not only effective but also respectful and inclusive across various cultural backgrounds.

Finally, the field would benefit from further research that delves deeper into the nuances of how interprofessional communication impacts medication safety. Longitudinal studies could help establish causality more definitively, and cross-cultural studies could shed light on how different healthcare systems and cultural contexts influence communication practices and their outcomes.

## Conclusions

Our investigation significantly contributes to the ongoing discourse surrounding patient safety, revealing the diverse elements that either enhance or hinder effective interprofessional communication within healthcare settings. While aligning with previous research, our analysis also uncovers significant insights into the complex dynamics of communication among healthcare professionals and within different operational environments. Specifically, our findings highlight the profound impact of organizational culture on communication effectiveness, demonstrating that environments fostering transparency and constructive feedback significantly reduce the occurrence of medication errors. Additionally, our research reveals a nuanced challenge related to the integration of digital communication tools. While these tools are essential for optimizing information exchange, they can sometimes lead to information overload, complicating communication processes. These insights underscore the importance of adopting a comprehensive and thoughtful approach to developing interventions aimed at reducing medication errors. Such an approach should integrate structural, educational, and policy-driven strategies while carefully considering the interplay of organizational culture and technological advancements.

## Figures and Tables

**Figure 1:**
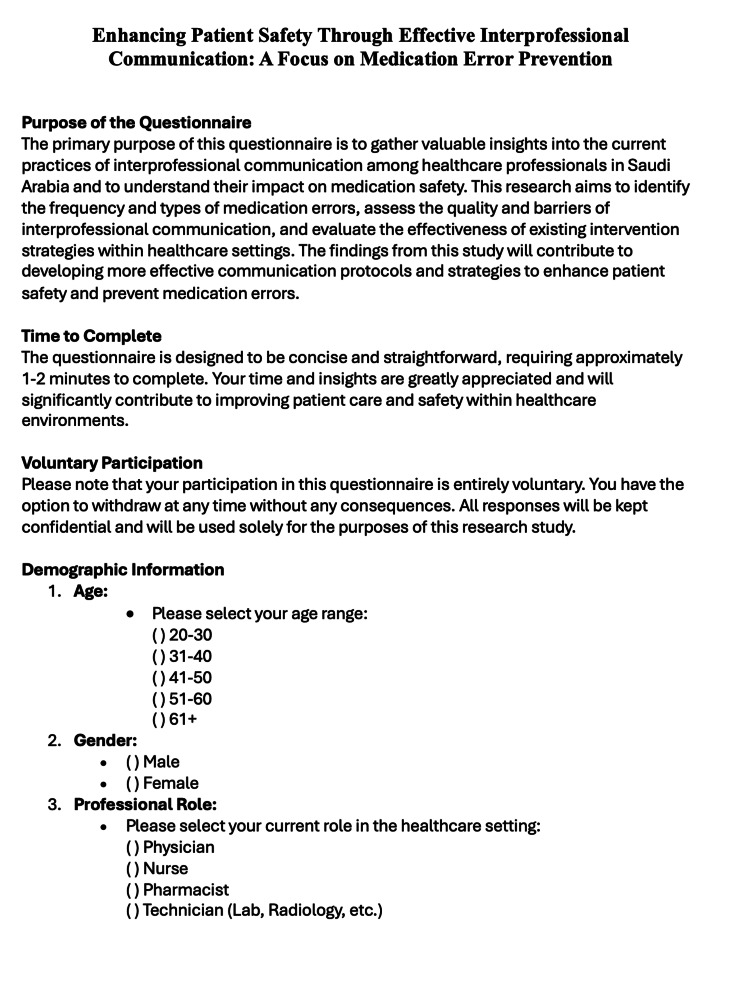
Questionnaire on interprofessional communication and medication error prevention in healthcare settings

**Figure 2:**
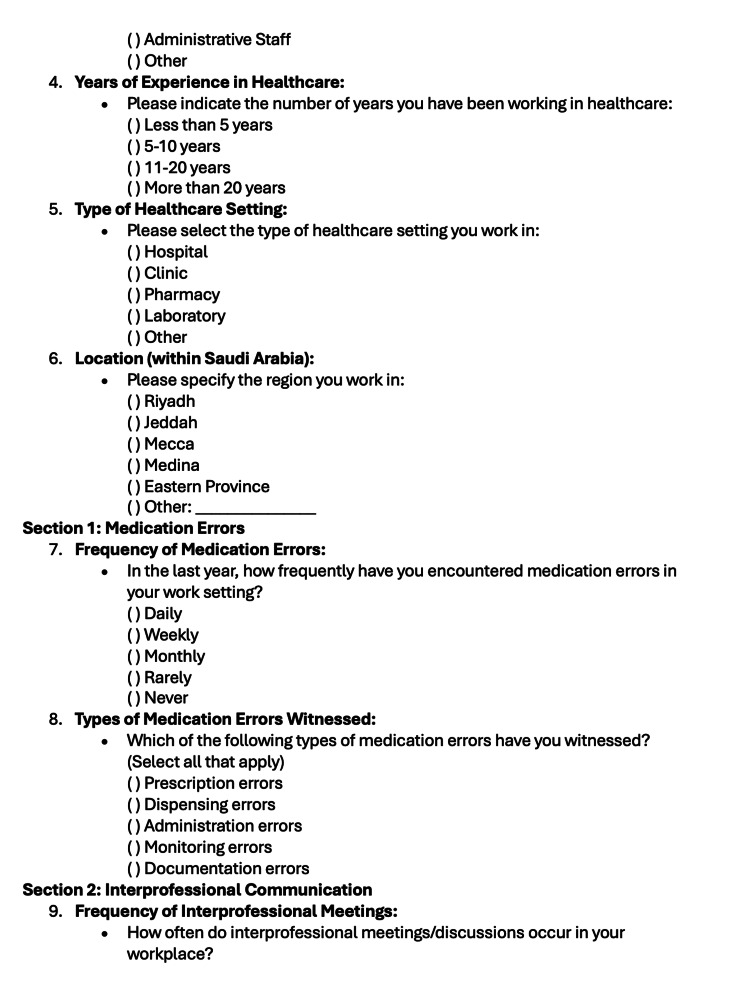
Questionnaire on interprofessional communication and medication error prevention in healthcare settings

**Figure 3:**
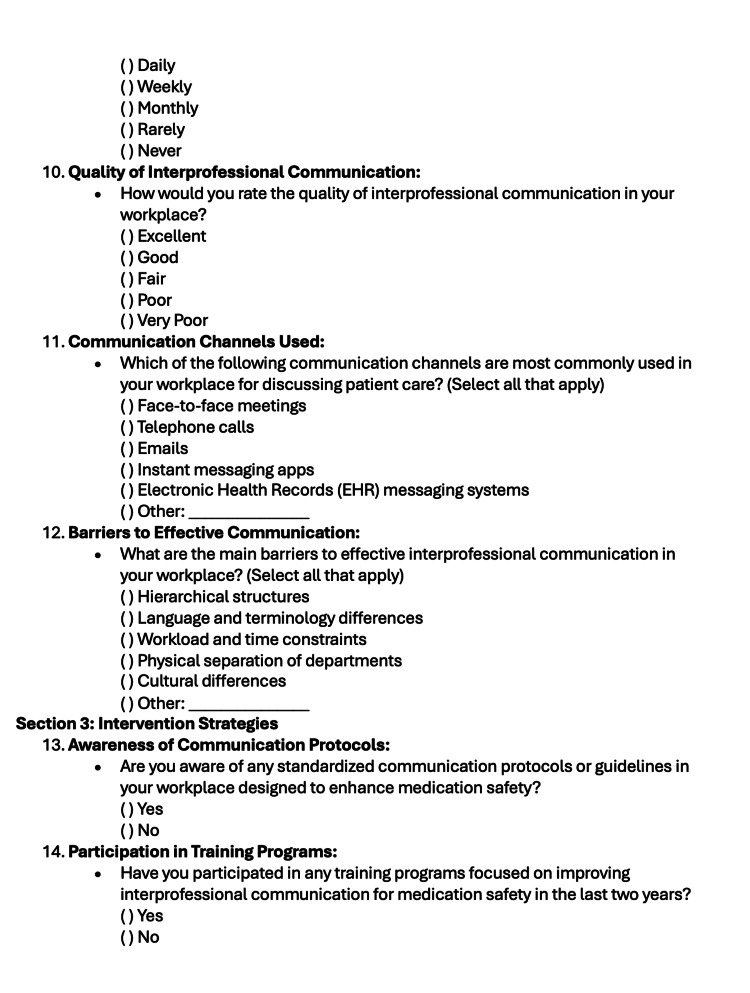
Questionnaire on interprofessional communication and medication error prevention in healthcare settings

**Figure 4:**
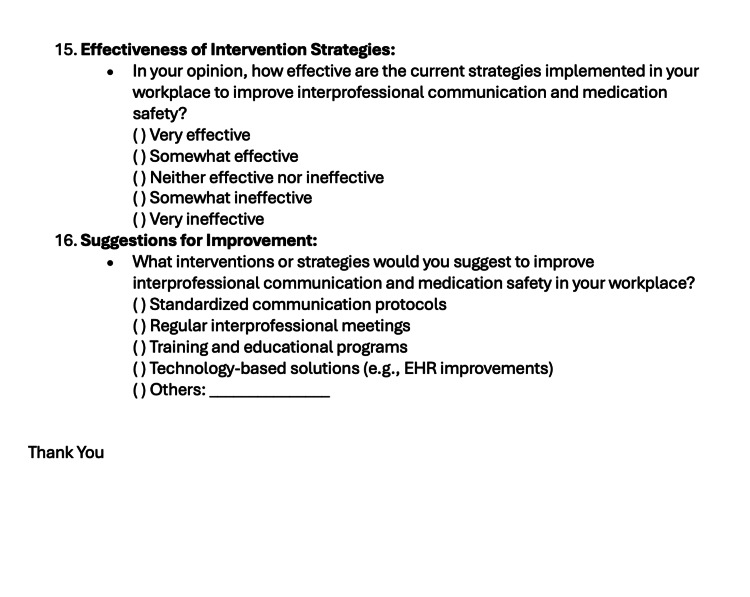
Questionnaire on interprofessional communication and medication error prevention in healthcare settings
